# Clinicopathological and prognostic value of hypoxia-inducible factor-1α in patients with bone tumor: a systematic review and meta-analysis

**DOI:** 10.1186/s13018-019-1101-5

**Published:** 2019-02-19

**Authors:** Deqing Luo, Hongyue Ren, Wenjiao Zhang, Hang Xian, Kejian Lian, Hui Liu

**Affiliations:** 1 Department of Orthopaedic Surgery, The Affiliated Southeast Hospital of Xiamen University, Orthopaedic Center of People’s Liberation Army, Zhangzhou, 363000 Fujian Province China; 2 Department of Pathology, The Affiliated Southeast Hospital of Xiamen University, Orthopaedic Center of People’s Liberation Army, Zhangzhou, 363000 Fujian Province China

**Keywords:** Hypoxia-inducible factor-1α, Bone tumor, Prognosis, Clinicopathological characteristics, Meta-analysis

## Abstract

**Background:**

Recently, many studies have shown the role of hypoxia-inducible factor-1α (HIF-1α) expression in the outcome of bone tumor. However, the results remain inconclusive. It is necessary to carry out a meta-analysis of all the current available data to clarify the relationship between HIF-1α and survival or clinicopathological features of bone tumor.

**Methods:**

PubMed, Cochrane Library, Web of Science, China National Knowledge Internet, and Wanfang databases were used to search the relationship between HIF-1α and bone tumor. Articles investigating clinicopathological and prognostic value of HIF-1α in bone tumor patients were enrolled in this meta-analysis. Overlapping articles, duplicate data, reviews, case reports, and letters without original data were excluded. The pooled risk ratios (RRs) and hazard ratios (HRs) were used to evaluate the clinicopathological and prognostic value of HIF-1α on bone tumor patients, respectively.

**Results:**

A total of 28 studies including 1443 patients were included in this meta-analysis, which were involved in three different types of bone tumor including 3 chondrosarcomas, 2 giant cell tumors of bone, and 23 osteosarcomas. Our results showed that high expression levels of HIF-1α were associated with poorer OS (overall survival) (HR = 2.61, 95% CI 2.11–3.23, *P* <  0.001) and shorter DFS (disease-free survival) (HR = 2.02, 95% CI 1.41–2.89, *P* <  0.001) in bone tumor. In addition, this study also analyzed the role of HIF-1α expression in clinicopathological features, which were closely related with the severity of bone tumor, including differentiation, clinical stage, metastasis, and microvessel density. Our results indicated that HIF-1α overexpression was significantly associated with differentiation (RR = 1.56, 95% CI 1.00–2.43, *P* = 0.049), clinical stage (RR = 1.75, 95% CI 1.25–2.45, *P* = 0.001), metastasis (RR = 1.78, 95% CI 1.58–2.00, *P* <  0.001), and microvessel density (SMD = 2.34, 95% CI 1.35–3.34, *P* <  0.001) of bone tumor.

**Conclusions:**

HIF-1α overexpression indicated an unfavorable factor for OS and DFS in bone tumor, suggesting that HIF-1α may serve as a potential prognostic marker for bone tumor.

**Electronic supplementary material:**

The online version of this article (10.1186/s13018-019-1101-5) contains supplementary material, which is available to authorized users.

## Background

Bone tumors are the fourth leading cause of cancer death in patients under 20 years, mainly presented as osteosarcoma, chondrosarcoma, Ewing sarcoma, giant cell tumor of bone, and fibrosarcoma [1]. Among them, osteosarcoma is the most common type of bone tumors, which often occurs in the distal femur and proximal tibia and commonly metastasizes to the lung. Although the 5-year survival rate of osteosarcoma patients has increased to 65–75% due to the improvement of surgical technology, chemotherapy, and radiotherapy [2], the survival rate of osteosarcoma patients with lung metastases remains just 28% [3]. Chondrosarcoma is a type of malignant bone tumors arising from tumor cells which produce cartilage matrix [4]. At present, surgery is the only curative treatment for patients with chondrosarcoma, because chondrosarcoma cells hardly respond to chemotherapy and radiation [5]. Giant cell tumor of bone is a benign bone tumor or sometimes semi-malignant neoplasm commonly affecting the knee [6]. Due to bone tumors appear mostly in children and adolescents, they will have a long-term impact on the life quality of patients [7]. Hence, it is necessary to explore the mechanism of the development and progression in bone tumor.

Hypoxia is an essential feature of many solid cancers and related to malignant transformation, metastases, and chemotherapy resistance [8, 9]. Hypoxia-inducible factors (HIFs) have severed as the critical molecular mediators in response to hypoxia. The HIF-1 is a heterodimer composed of HIF-1α and HIF-1β subunits. HIF-1β is constitutively expressed while HIF-1α is regulated according to oxygen concentration [10]. Under normoxic condition, HIF-1α is rapidly degraded by Von Hippel-Lindau (VHL) through the ubiquitin-proteasomal pathway. Under hypoxia condition, the degradation process is inhibited and HIF-1α transfers from the cell plasma to the nucleus, where it can bind to hypoxia-response elements (HREs) regulating the transcription of many genes relevant to oxygen transport, glucose metabolism, cell proliferation, and apoptosis [11, 12]. A large amount of studies have paid close attention to the expression of HIF-1α in the prognosis of various cancers including breast cancer, esophageal squamous cell carcinoma, hepatocellular carcinoma, gastric cancer, and lung cancer [13–16]. Furthermore, HIF-1α plays an important role in the bone tumor involved in pivotal aspects of tumor biology [17]. However, the clinicopathological and prognostic value of HIF-1α has been controversial in patients with bone tumor [18, 19].

In order to clarify the effect of HIF-1α on the clinicopathological and prognostic value in the bone tumor, a meta-analysis was performed to systematically evaluate the relationship between HIF-1α expression and bone tumor.

## Materials and methods

This study was performed totally following the guidelines of the Preferred Reporting Items for Systematic Reviews and Meta-Analyses (PRISMA) [20].

### Search strategy

The PubMed (MEDLINE), Cochrane Library, Web of Science together with two Chinese databases, China National Knowledge Internet (CNKI) and Wanfang databases, were used to search for articles that evaluated the role of HIF-1α in the prognosis of bone tumor. Studies eligible for this analysis were updated on May 5, 2018, using the search terms “hypoxia-inducible factor-1”, “HIF-1”, and “bone tumor”, “bone sarcoma”, “osteosarcoma”, “chondrosarcoma”, “Ewing sarcoma”, “giant cell tumor”.

### Criteria for inclusion and exclusion

The inclusion criteria are as follows: (1) articles investigating the relation between HIF-1α and bone tumor patients, (2) the HIF-1α expression in bone tumor tissues were detected, (3) patients were grouped according to the expression levels of HIF-1α, and (4) related clinicopathological features were described. The exclusion criteria are as follows: (1) literatures not pertinent to the HIF-1α, (2) studies not relevant to HIF-1α expression or lack of survival outcome and clinicopathological features, (3) overlapping articles or duplicate data, and (4) reviews, case reports, and letters without original data.

### Data collection

Deqing Luo and Hongyue Ren screened the full text of selected studies to confirm eligibility and then extracted data independently. Disagreement was dissolved by consulting with a third author (Hui Liu). For each study, the following information was recorded: first author, publication year, country, histological type, the patient’s cases, the number of HIF-1α positive, the percentage of HIF-1α positive, survival, and follow-up time. The Newcastle-Ottawa Scale (NOS) score was used for assessing the quality, since all the included studies were non-randomized and retrospective studies. Studies with scores of 5 to 9 were regarded as high quality; otherwise, those with scores of zero to four were considered as low quality.

### Statistical analysis

All analyses were performed using STATA 12 software (STATA Corp., College Station, TX). The effects of HIF-1α expression on outcome of the bone tumor were described as hazard ratios (HRs) with an estimate of 95% confidence intervals (CIs), which directly obtain from the publication or retrieve from Kaplan-Meier Curves by extracting several survival rates at specified times from the curves. The intensity of relationship between HIF-1α expression and clinicopathological features was assessed by risk ratios (RRs) and corresponding 95% CIs. Continuous data were expressed as standard mean difference (SMD) with 95% CIs. The chi-square-based Cochrane’s *Q* test and *I*^2^ index were conducted to evaluate the study of heterogeneity. If there was mild heterogeneity among studies (*P* > 0.10, *I*^2^ <  50%), the fixed effects model was applied to pooled data; otherwise, the random effects model was used (*P* <  0.10, *I*^2^ > 50%). Publication bias was calculated by Begg’s funnel plot and Egger’s test. Sensitivity analysis was used to evaluate the stability of the results by omitting individual study sequentially. A *P* value less than 0.05 was defined as statistical significance.

## Results

### Characteristics of studies

**Fig. 1:**
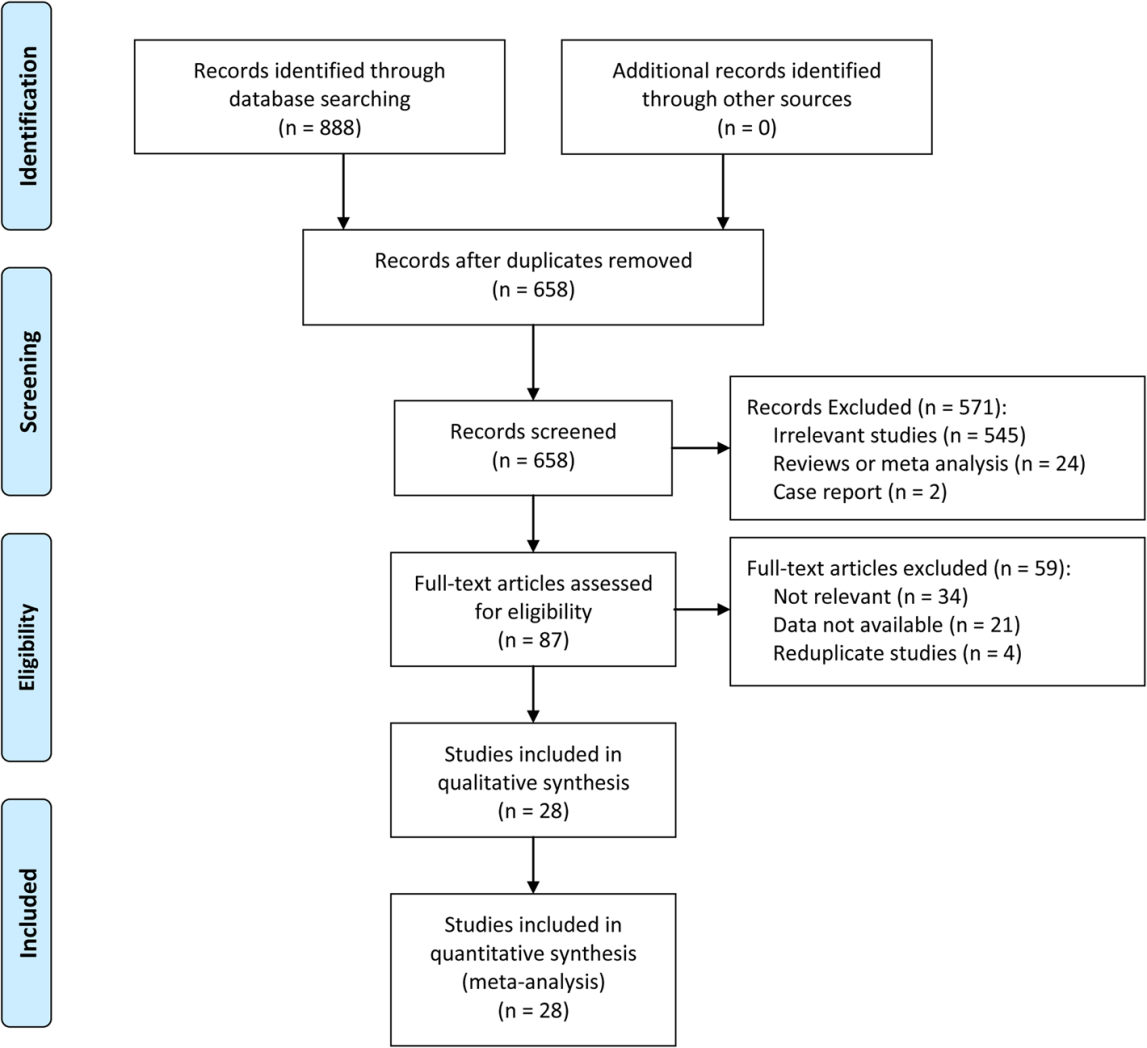
The flow diagram of this meta-analysis

As shown in Fig. 1, a total of 888 studies were retrieved on initial literature search that related to the clinicopathological and prognostic value of HIF-1α in patients with bone tumor. Finally, after scanning the titles, abstracts, and full texts, 28 articles were included in the current meta-analysis [21–48]. The main and clinicopathological characteristics of the included studies were summarized in Table 1 and Additional file 1: Table S1, respectively. All studies were published between 2008 and 2017 with a total of 1443 patients from Germany, Japan, Canada, and China. Out of the 28 studies, 22 studies evaluated the relationship between HIF-1α expression and the clinicopathological features of bone tumor, and 14 studies reported survival data.

**Table 1:** Main characteristics of included studies in the meta-analysis

References	Year	Country	Histological type	Case (*n*)	HIF-1α + (*n*)	HIF-1α + (%)	Survival	Follow-up (months)	NOS score	Quality
Bao [21]	2013	China	Osteosarcoma	108	62	57	OS	60	4	Low
Boeuf [22]	2010	Germany	Chondrosarcoma	32	14	44	DFS	100	4	Low
Chen [23]	2008	China	Osteosarcoma	25	17	68	NR	NR	3	Low
Chen [24]	2010	China	Chondrosarcoma	34	20	59	OS	100	4	Low
Chen [25]	2012	China	Osteosarcoma	49	27	55	DFS	100	6	High
Geng [26]	2008	China	Osteosarcoma	59	23	39	OS	50	6	High
Guan [27]	2014	China	Osteosarcoma	52	46	88	NR	NR	3	Low
Guo [28]	2014	China	Osteosarcoma	98	78	80	OS	120	4	Low
Hu [29]	2009	China	Osteosarcoma	35	23	66	NR	NR	3	Low
Hu [30]	2015	China	Osteosarcoma	50	29	58	DFS	50	4	Low
Kubo [31]	2008	Japan	Chondrosarcoma	20	8	40	DFS	120	4	Low
Li [32]	2012	China	Osteosarcoma	102	47	46	NR	NR	3	Low
Li [33]	2015	China	Osteosarcoma	28	15	54	NR	NR	4	Low
Lian [34]	2013	China	Osteosarcoma	35	12	34	NR	NR	3	Low
Luo [35]	2009	China	GCTB	51	32	63	NR	NR	4	Low
Ma [36]	2014	China	GCTB	80	40	50	NR	NR	4	Low
Mao [37]	2007	China	Osteosarcoma	64	37	58	NR	NR	4	Low
Mizobuchi [38]	2008	USA	Osteosarcoma	48	18	38	NR	NR	3	Low
Naggar [39]	2012	Canada	Osteosarcoma	25	13	52	NR	NR	3	Low
Qian [40]	2007	China	Osteosarcoma	25	14	56	NR	NR	4	Low
Wang [41]	2004	China	Osteosarcoma	46	16	35	OS	70	5	High
Wang [42]	2017	China	Osteosarcoma	103	57	55	OS	120	7	High
Wu [43]	2010	China	Osteosarcoma	36	15	42	NR	NR	3	Low
Yang [44]	2007	China	Osteosarcoma	39	31	79	OS/DFS	100	6	High
Yin [45]	2010	China	Osteosarcoma	36	22	61	OS	36	5	High
Zeng [46]	2010	China	Osteosarcoma	45	25	56	OS	60	6	High
Zhao [47]	2015	China	Osteosarcoma	88	50	57	OS	107	7	High
Zheng [48]	2009	China	Osteosarcoma	30	15	50	NR	NR	4	Low

*HIF-1α +* hypoxia-inducible factor-1α expression, *GCTB* giant cell tumor of bone, *OS* overall survival, *DFS* disease-free survival, *NR* not reported, *NOS* Newcastle–Ottawa scale

Three different types of bone tumor were involved in this meta-analysis, with 3 chondrosarcomas, 2 giant cell tumors of bone and 23 osteosarcomas. Sample sizes ranged from 20 to 108 cases (mean sample size, 52). Overall, the overall survival (OS) or disease-free survival (DFS) of these studies were ranged from 50 to 120 months, and 8 studies were evaluated to be of high quality.

### Correlation between HIF-1α and the clinicopathological features

**Fig. 2:**
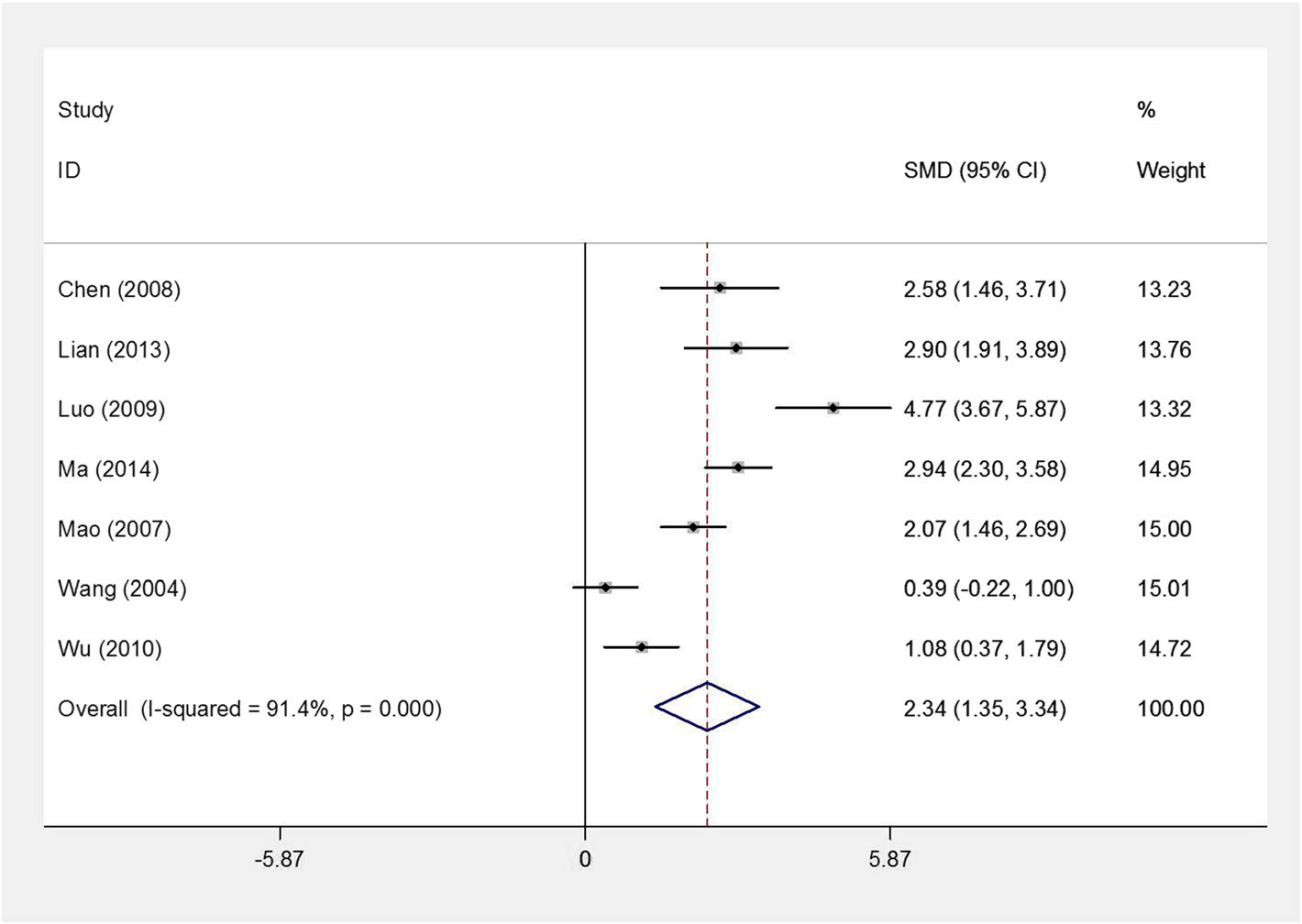
Forest plots of the association between HIF-1α expression and microvessel density (MVD) in patients with bone tumor

To explore the relationship between HIF-1α expression and the clinicopathological factors of patients with bone tumor, the analyses were conducted to stratify by gender, age, tumor size, differentiation, clinical stage, metastasis, and microvessel density (MVD). Among them, clinicopathologic factors, including tumor differentiation, clinical stage, metastasis, and MVD of bone tumor, are closely related with the malignant level of bone tumor and also used to estimate the prognosis of patients with bone tumor. As shown in Table 2 and Additional file 2: Figure S1, HIF-1α expression did not show any significant association with gender (RR = 0.93, 95% CI 0.83–1.04, *P* = 0.179; fixed effects model: *χ*^2^ = 12.97, *I*^2^ = 7.5, *P* = 0.371), age (RR = 1.19, 95% CI 1.00–1.41, *P* = 0.055; fixed effects model: *χ*^2^ = 8.72, *I*^2^ = 42.7, *P* = 0.121), and tumor size (RR = 1.19, 95% CI 0.99–1.44, *P* = 0.069; fixed effects model: *χ*^2^ = 6.86, *I*^2^ = 12.6, *P* = 0.334). However, high HIF-1α expression was related with tumor differentiation (RR = 1.56, 95% CI 1.00–2.43, *P* = 0.049; random effects model: *χ*^2^ = 28.33, *I*^2^ = 75.3, *P* <  0.001), clinical stage (RR = 1.75, 95% CI 1.25–2.45, *P* = 0.001; fixed effects model: *χ*^2^ = 8.89, *I*^2^ = 43.8, *P* = 0.113), and metastasis (RR = 1.78, 95% CI 1.58–2.00, *P* <  0.001; fixed effects model: *χ*^2^ = 24.91, *I*^2^ = 47.8, *P* = 0.024). In addition, as shown in Fig. 2, high HIF-1α expression was strongly associated with MVD of bone tumor (SMD = 2.34, 95% CI 1.35–3.34, *P* <  0.001; random effects model: *χ*^2^ = 69.97, *I*^2^ = 91.4, *P* <  0.001).

**Table 2:** The analysis for HIF-1α and the clinicopathological features of patients with bone tumor

Clinicopathological features	Number of studies	Number of case (*n*)	Number of HIF-1α + (*n*)	Pooled data			Test for heterogeneity		
				RR	95% CI	*P* value	*χ* ^2^	*P* value	*I*^2^ (%)
Gender (male vs. female)	13	832	500	0.93	0.83–1.04	0.179	12.97	0.371	7.5
Age (year) (≤ 20 vs. > 20)	6	481	303	1.19	1.00–1.41	0.055	8.72	0.121	42.7
Size (cm) (≥ 5.0 vs. < 5)	7	378	222	1.19	0.99–1.44	0.069	6.86	0.334	12.6
Differentiation (poor vs. well/moderate)	8	272	174	1.56	1.00–2.43	0.049	28.33	< 0.001	75.3
Clinical stage (II/III vs. I)	6	396	207	1.75	1.25–2.45	0.001	8.89	0.113	43.8
Metastasis (yes vs. no)	14	870	503	1.78	1.58–2.00	< 0.001	24.91	0.024	47.8

*HIF-1α +* hypoxia-inducible factor-1α expression, *RR* risk ratio, *CI* confidence interval

### Association between HIF-1α and prognosis in patients with bone tumor

**Fig. 3:**
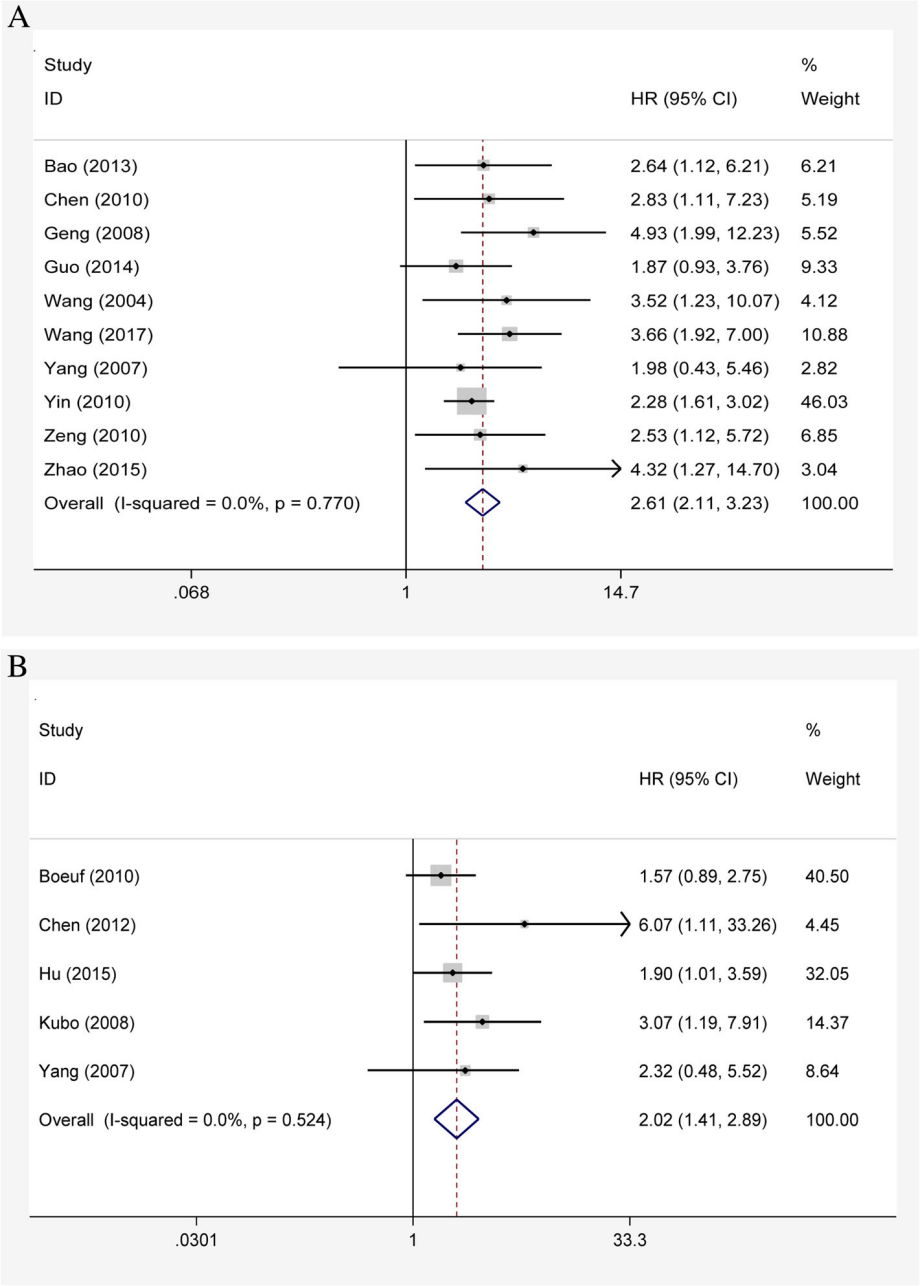
Forest plots of the association between HIF-1α expression and overall survival (OS) (**a**) or disease-free survival (DFS) (**b**) in patients with bone tumor

**Fig. 4:**
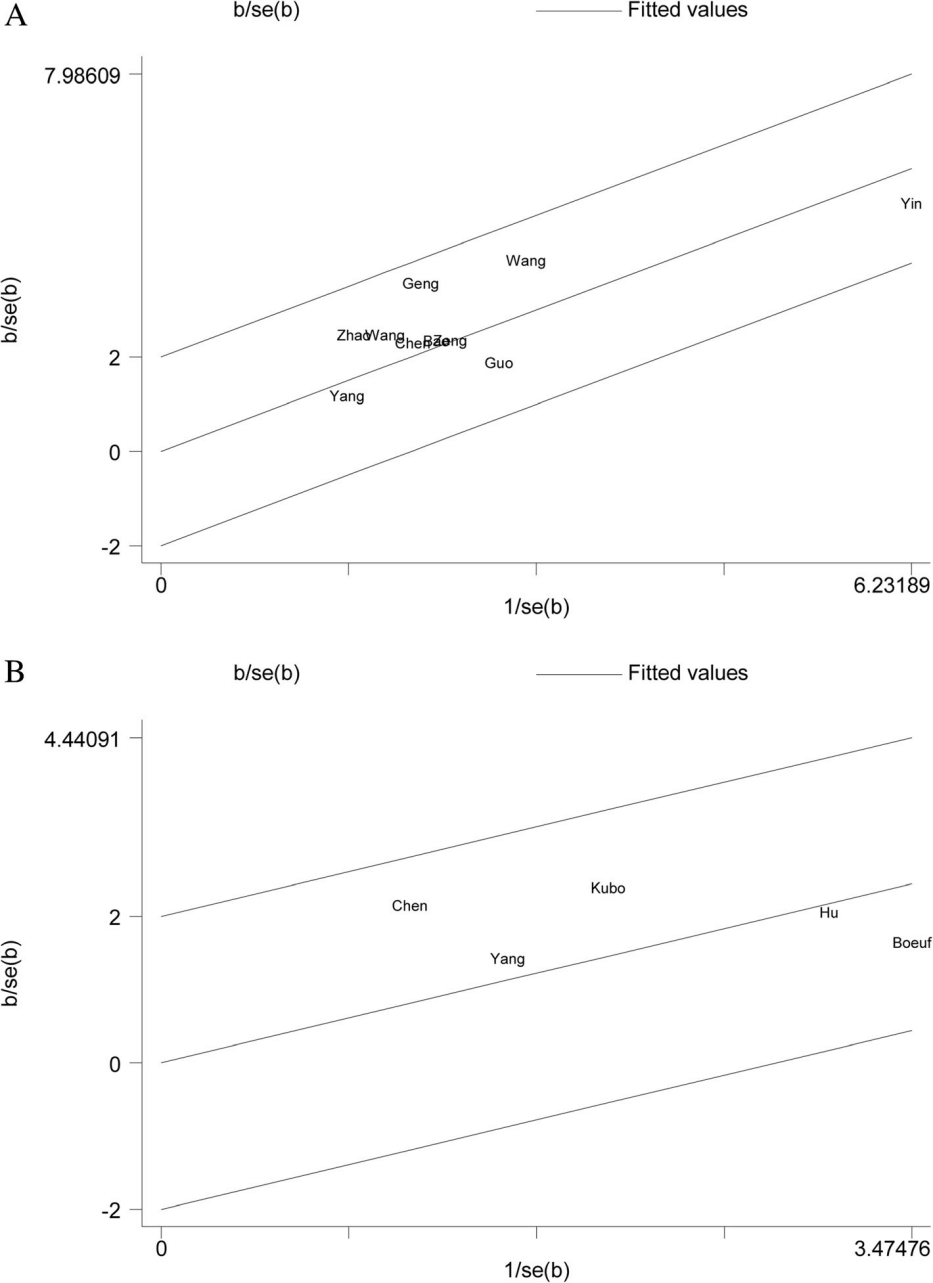
Galbraith plot analysis of the effect of HIF-1α expression on overall survival (OS) (**a**) or disease-free survival (DFS) (**b**)

A total of ten studies have assessed the association of HIF-1α expression with OS. As shown in Fig. 3a, high expression level of HIF-1α significantly predicted unfavorable OS in bone tumor (HR = 2.61, 95% CI 2.11–3.23, *P* <  0.001), without any heterogeneity in the data (fixed effects model: *χ*^2^ = 5.70, *I*^2^ = 0, *P* = 0.770). Correspondingly, Galbraith graph also showed no heterogeneity in this meta-analysis (Fig. 4a). Furthermore, we investigated the relationship between HIF-1α expression and DFS of bone tumor. As shown in Fig. 3b, the combined data of five studies provided information on DFS demonstrated that patients with HIF-1α overexpression had shorter DFS (HR = 2.02, 95% CI 1.41–2.89, *P* <  0.001; fixed effects model: *χ*^2^ = 3.21, *I*^2^ = 0, *P* = 0.524). Correspondingly, it also did not show heterogeneity in the Galbraith graph (Fig. 4b).

### Subgroup analyses

To further explore the relationship between HIF-1α expression and the prognosis of patients with bone tumor, subgroup analyses of OS (Table 3 and Additional file 3: Figure S2) and DFS (Table 4 and Additional file 4: Figure S3) were performed to stratify by region, histological type, the number of cases, follow-up time, or the quality of included articles. For OS, in the subgroup analysis based on histological type, the results showed that HIF-1α overexpression existed poor OS in osteosarcoma (HR = 2.60, 95% CI 2.09–3.24, *P* <  0.001) and chondrosarcoma (HR = 2.83, 95% CI 1.11–7.22, *P* = 0.030). The relation between HIF-1α overexpression and the OS of patients with bone tumor was also present in studies with less than 100 months (HR = 2.55, 95% CI 1.97–3.30, *P* <  0.001) as well as more than or equal 100 months follow-up time (HR = 2.76, 95% CI 1.88–4.04, *P* <  0.001). In addition, HIF-1α overexpression showed poor OS in the studies with smaller cases (*n* <  50) (HR = 2.40, 95% CI 1.84–3.12, *P* <  0.001), larger cases (*n* ≥ 50) (HR = 3.07, 95% CI 2.14–4.40, *P* <  0.001), high quality (HR = 2.70, 95% CI 2.13–3.43, *P* <  0.001), and low quality (HR = 2.30, 95% CI 1.44–3.68, *P* <  0.001).

**Table 3:** The subgroups analysis for HIF-1α and OS in patients with bone tumor

Subgroups	Number of studies	Case (*n*)	HIF-1α + (*n*)	HIF-1α + (%)	Pooled data			Test for heterogeneity	
					HR	95% CI	*P* value	*P* value	*I*^2^ (%)
Histological type									
Osteosarcoma	9	622	364	59	2.60	2.09–3.24	< 0.001	0.684	0
Chondrosarcoma	1	34	20	59	2.83	1.11–7.22	0.030	NA	NA
Case (*n*)									
< 50	5	200	114	57	2.40	1.84–3.12	< 0.001	0.770	0
≥ 50	5	456	270	59	3.07	2.14–4.40	< 0.001	0.451	0
Follow-up (months)									
< 100	5	294	184	50	2.55	1.97–3.30	< 0.001	0.578	0
≥ 100	5	362	236	65	2.76	1.88–4.04	< 0.001	0.608	0
Quality									
High	7	416	224	54	2.70	2.13–3.43	< 0.001	0.581	0
Low	3	240	160	67	2.30	1.44–3.68	< 0.001	0.732	0

*OS* overall survival, *HIF-1α +* hypoxia-inducible factor-1α expression, *HR* hazard ratio, *CI* confidence interval, *NA* not available due to single study

**Table 4:** The subgroups analysis for HIF-1α and DFS in patients with bone tumor

Subgroups	Number of studies	Case (*n*)	HIF-1α + (*n*)	HIF-1α + (%)	Pooled data			Test for heterogeneity	
					HR	95% CI	*P* value	*P* value	*I*^2^ (%)
Region									
Non-Asian	2	52	22	42	1.87	1.15–3.04	0.011	0.233	29.7
Asian	3	138	87	63	2.21	1.30–3.78	0.004	0.454	0
Histological type									
Osteosarcoma	3	138	87	63	2.21	1.30–3.78	0.004	0.454	0
Chondrosarcoma	2	52	22	42	1.87	1.15–3.04	0.011	0.233	29.7
Case (*n*)									
< 40	3	91	53	58	1.93	1.23–3.02	0.004	0.467	0
≥ 40	2	99	56	57	2.19	1.21–3.97	0.010	0.210	36.4
Follow-up (months)									
< 100	1	50	29	58	1.90	1.01–3.58	0.047	NA	NA
≥ 100	4	140	80	57	2.08	1.34–3.21	0.001	0.368	5
Quality									
High	2	88	58	66	3.22	1.19–8.68	0.021	0.368	0
Low	3	102	51	50	1.88	1.28–2.77	0.001	0.491	0

*DFS* disease-free survival, *HIF-1α +* hypoxia-inducible factor-1α expression, *HR* hazard ratio, *CI* confidence interval, *NA* not available due to single study

In the subgroup for HIF-1α expression and DFS, HIF-1α overexpression showed poor DFS in the non-Asian (HR = 1.87, 95% CI 1.15–3.04, *P* = 0.011), the Asian regions (HR = 2.21, 95% CI 1.30–3.78, *P* = 0.004), osteosarcoma (HR = 2.21, 95% CI 1.30–3.78, *P* = 0.004), and chondrosarcoma (HR = 1.87, 95% CI 1.15–3.04, *P* = 0.011). Furthermore, HIF-1α overexpression showed shorter DFS in the studies with smaller cases (*n* < 40) (HR = 1.93, 95% CI 1.23–3.02, *P* = 0.004), larger cases (*n* ≥ 40) (HR = 2.19, 95% CI 1.21–3.97, *P* = 0.010), long follow-up time (≥ 100) (HR = 2.08, 95% CI 1.34–3.21, *P* = 0.001), and short follow-up time (< 100) (HR = 1.90, 95% CI 1.01–3.58, *P* = 0.047), high quality (HR = 3.22, 95% CI 1.19–8.68, *P* = 0.021), and low quality (HR = 1.88, 95% CI 1.28–2.77, *P* = 0.001).

### Sensitivity analyses and publication bias

**Fig. 5:**
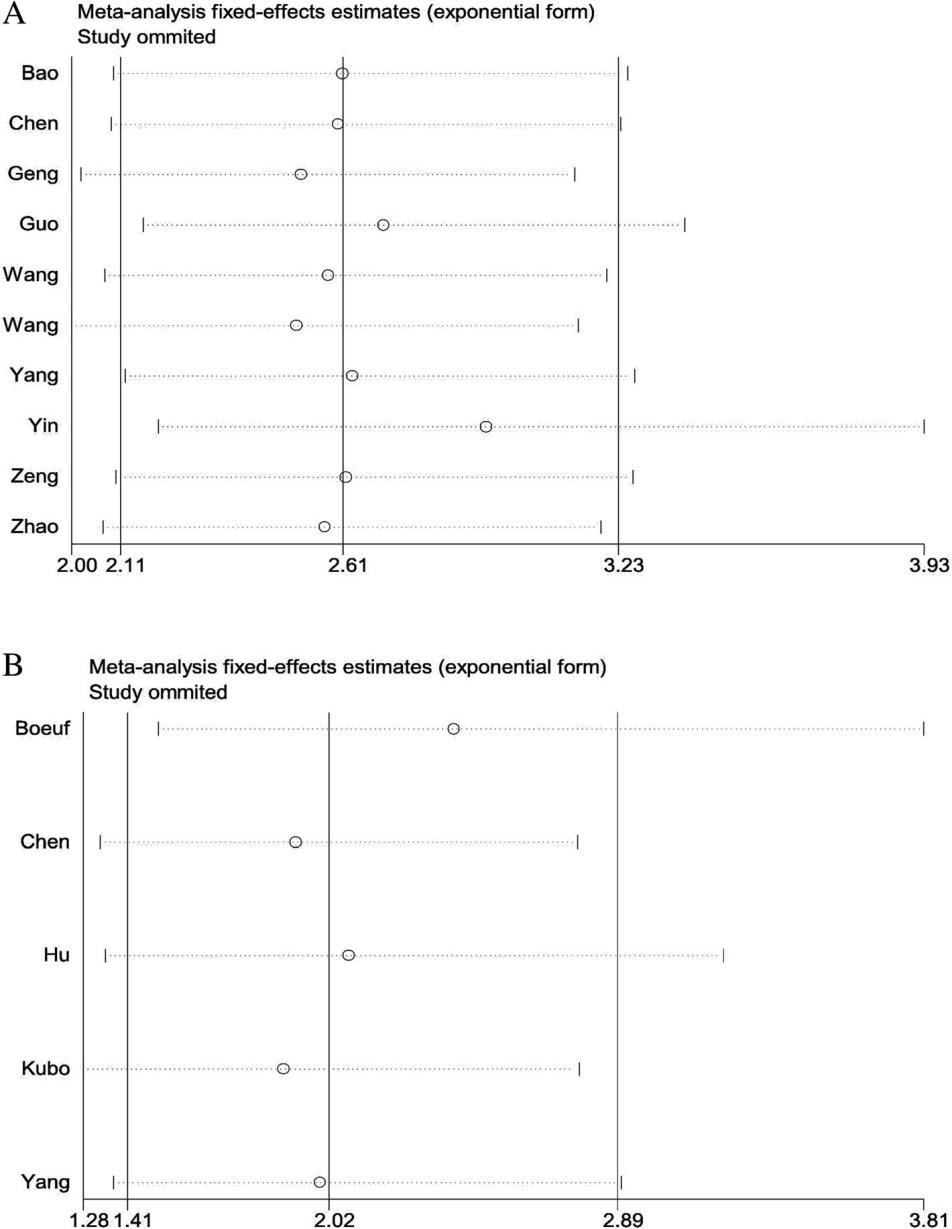
Sensitivity analysis of the effect of HIF-1α expression on overall survival (OS) (**a**) or disease-free survival (DFS) (**b**)

**Fig. 6:**
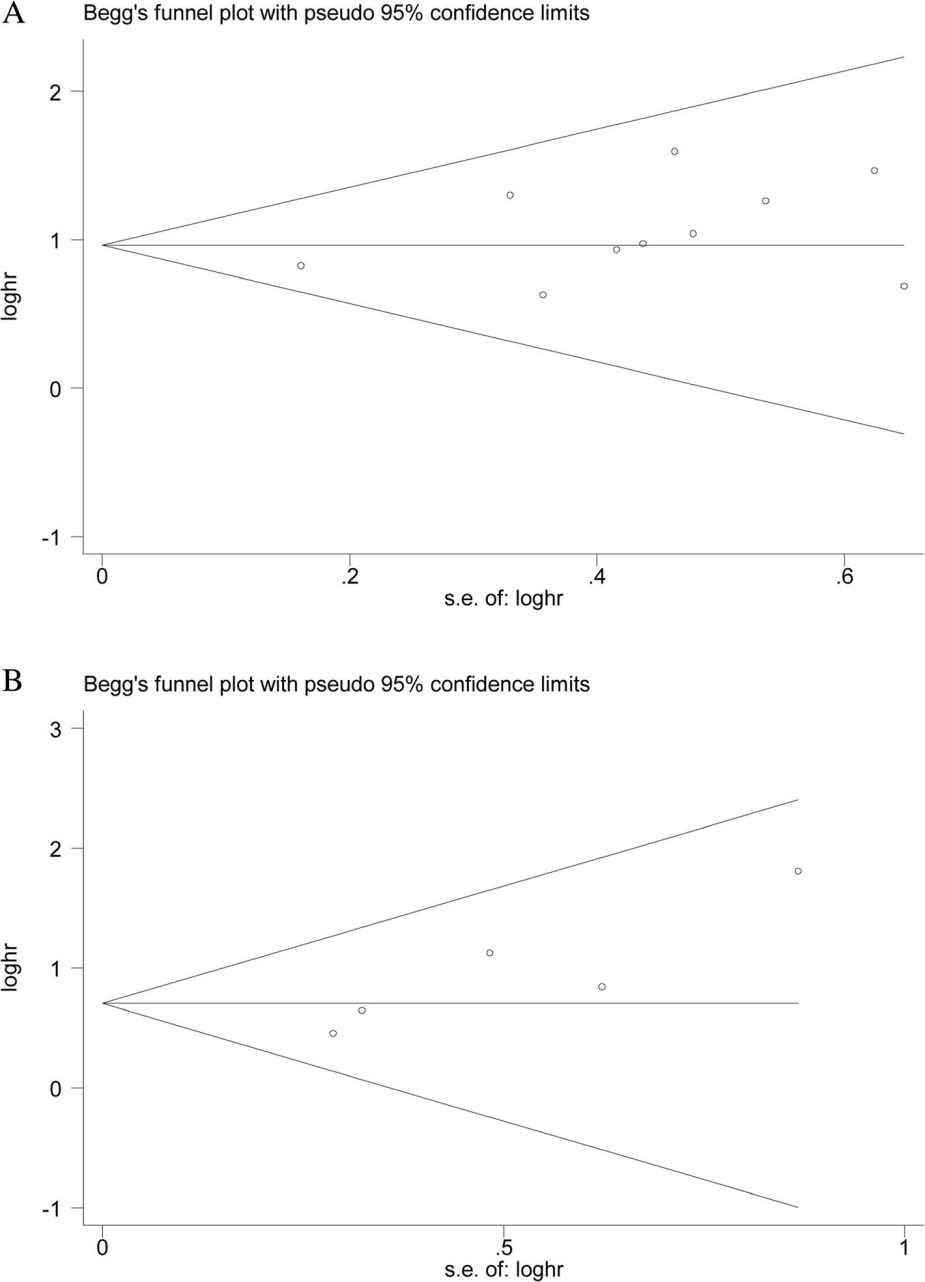
Begg’s funnel plot of the effect of HIF-1α expression on overall survival (OS) (**a**) or disease-free survival (DFS) (**b**)

Sensitivity analysis was conducted to evaluate the effect of any single study on the prognosis. No significant difference was found after a sequential omission of one study at a time, suggesting that the conclusions of OS (Fig. 5a) and DFS (Fig. 5b) were stable. In addition, publication bias of the included literatures was performed to assess by Begg’s plot and Egger’s tests. As shown in Fig. 6a, the tests revealed that no evidence of publication bias in the analysis of OS (Begg’s *P* = 0.283 and Egger’s *P* = 0.150). In addition, there was no obvious evidence of publication bias on DFS assessed by Begg’s tests; however, Egger’s tests indicated a significant publication bias on DFS (Begg’s *P* = 0.086 and Egger’s *P* = 0.037) (Fig. 6b).

## Discussion

HIF-1α is an important regulator of cellular response to hypoxia. Increased expression of HIF-1α, a marker of tumor hypoxia, is well associated with carcinogenesis and tumor progression in various kinds of cancer [49]. Recent studies have shown that overexpression of HIF-1α was linked with unfavorable prognosis in some malignancies [50]. A meta-analysis indicated no significant relationship between HIF-1α and the DFS of osteosarcoma [18]. However, a recent study found that HIF-1α might play an important role in the evolution of osteosarcoma [19]. Based on the aforementioned controversy, we conducted this meta-analysis to evaluate the role of HIF-1α in the prognosis and clinicopathological features of patients with bone tumor.

In this meta-analysis, a total of 888 studies with 1443 patients were obtained. The pooled results showed that HIF-1α overexpression was significantly associated with poorer OS and shorter DFS in patients with bone tumor. Moreover, significant results were also found in the subgroup analyses of OS and DFS by region, histological type, the number of cases, follow-up time, and the quality. What was more, HIF-1α overexpression was also significantly associated with the differentiation, clinical stage, metastasis, and MVD of bone tumor. Among them, tumor differentiation represents the severity of the bone tumor. Clinical stage of bone tumor is related to the prognosis and outcome of patients. The main reason for failure treatment of bone tumors is metastasis, which often depends on tumor angiogenesis. MVD is an important indicator of bone tumor angiogenesis and is associated with the prognosis of bone tumor. Hence, our results may provide some implications for doctors in practice. In addition, the results of OS and DFS were stable. There was no obvious evidence of publication bias on OS. However, Egger’s tests indicated a significant publication bias on DFS, because there are only five studies included in this meta-analysis. Besides, Ewing sarcoma was also one of the frequent bone tumors, but it was not enrolled in this meta-analysis. In fact, we have searched several original articles, which investigated the relationship between HIF-1α and Ewing sarcoma [51, 52]. Unfortunately, these articles were excluded due to lack of prognosis (OS and DFS) and clinicopathological features of Ewing sarcoma. Further large studies with high quality are required.

As a key transcriptional regulator, HIF-1α has critical effect on the development and progression of tumor cells by activating the targeting genes, which can regulate several biological processes including cell proliferation, survival, migration, angiogenesis, and glucose metabolism. In addition, HIF-1α can also play a significant role in bone tumor. Increasing evidences have indicated that HIF-1α was not only expressed under normoxia in the osteosarcoma cell line [53], but also overexpressed in metastatic osteosarcoma tumors [38]. More importantly, HIF-1α can contribute to the proliferation, migration, and chemoresistance in osteosarcoma, chondrosarcoma, and giant cell tumor. There was evidence that hypoxia promoted migration of human osteosarcoma cells by activating the HIF-1α/CXCR4 pathway [54]. In chondrosarcoma, HIF-1α could promote the expression of vascular endothelial growth factor (VEGF), which was the primary cytokine related to angiogenesis [55]. Furthermore, increased expression levels of HIF-1α played a prominent role in evasion of apoptosis and chondrosarcoma progression through upregulation of Bcl-xl [56].

Although we evaluated comprehensively the association between HIF-1α and bone tumor outcome, there were several limitations in this meta-analysis. Firstly, Egger’s tests indicated a significant publication bias on DFS. It was possibly because positive results were more likely to be published than negative ones, and further large studies are required. Secondly, HRs were extracted from Kaplan-Meier curves in a few studies, which may not have been entirely accurate. Thirdly, most of the included patients with bone tumor were from China, researches from other countries might obtain different outcomes. Finally, all of the included articles were retrospective studies and most of them had small sample size.

In conclusion, this meta-analysis provides a strong evidence of the correlation of HIF-1α overexpression with both clinicopathological features and survival in patients with osteosarcomas, chondrosarcomas, and giant cell tumors of the bone, suggesting HIF-1α could be used as a useful biomarker for predicting the prognosis of bone tumor patients.

## Additional files

Additional file 1: **Table S1.** Clinicopathological characteristics of included studies in the meta-analysis. (DOCX 32 kb)

Additional file 2: **Figure S1.** Forest plots of the association between HIF-1α expression and the clinicopathological factors of patients with bone tumor including gender (A), age (B), tumor size (C), differentiation (D), clinical stage (E) and metastasis (F). (TIF 1328 kb)

Additional file 3: **Figure S2.** Forest plots of subgroup analyses on the association between HIF-1α expression and OS including histological type (A), the number of case (B), follow-up time (C) and the quality of included articles (D). (TIF 1145 kb)

Additional file 4: **Figure S3.** Forest plots of subgroup analyses on the association between HIF-1α expression and DFS including region (A), histological type (B), the number of case (C), follow-up time (D) and the quality of included articles (E). (TIF 1333 kb)

## Abbreviations

CI: Confidence intervals
CNKI: China National Knowledge Internet
DFS: Disease-free survival
HIF-1α: Hypoxia-inducible factor-1α
HREs: Hypoxia-response elements
HRs: Hazard ratios
MVD: Microvessel density
NOS: Newcastle-Ottawa Scale
OS: Overall survival
PRISMA: Preferred Reporting Items for Systematic Reviews and Meta-Analyses
RRs: Risk ratios
SMD: Standard mean difference
VHL: Von Hippel-Lindau
