# Supplementary material for: Clinicopathological and prognostic value of hypoxia-inducible factor-1α in patients with bone tumor: a systematic review and meta-analysis
Source: J Orthop Surg Res. 2019 Feb 19;14:56. doi: 10.1186/s13018-019-1101-5 (PMC6381668; doi:10.1186/s13018-019-1101-5)
Supplement: Supplementary file 1 — Table S1. Clinicopathological characteristics of included studies in the meta-analysis. (DOCX 32 kb) [file 13018_2019_1101_MOESM1_ESM.docx]

**Table S1** Clinicopathological characteristics of included studies in the meta-analysis.

| Study | Year | Case (n) | HIF-1α + (n) | HIF-1α expression | Gender | | Age (years) | | Size (cm) | | Differentiation | | Clinical stage | | Metastasis | | MVD |
| --- | --- | --- | --- | --- | --- | --- | --- | --- | --- | --- | --- | --- | --- | --- | --- | --- | --- |
|  |  |  |  |  | Male | Female | ≤ 20 | > 20 | ≥ 5 | < 5 | Poor | Well/Mediate | Ⅱ- Ⅲ | Ⅰ | Yes | No |  |
| Bao | 2013 | 108 | 62 | Positive | 36 | 26 | 37 | 27 | 28 | 34 | - | - | 56 | 6 | 40 | 22 | - |
|  |  |  |  | Negative | 31 | 15 | 20 | 26 | 20 | 26 | - | - | 29 | 17 | 19 | 27 | - |
| Boeuf | 2010 | 32 | 14 | Positive | - | - | - | - | - | - | - | - | - | - | - | - | - |
|  |  |  |  | Negative | - | - | - | - | - | - | - | - | - | - | - | - | - |
| Chen | 2008 | 25 | 17 | Positive | - | - | - | - | 14 | 3 | 3 | 14 | 14 | 3 | 6 | 11 | 45.96 ± 5.52 |
|  |  |  |  | Negative | - | - | - | - | 2 | 6 | 6 | 2 | 2 | 6 | 0 | 8 | 29.84 ± 7.63 |
| Chen | 2010 | 34 | 20 | Positive | 10 | 10 | - | - | - | - | 11 | 9 | - | - | - | - | - |
|  |  |  |  | Negative | 6 | 8 | - | - | - | - | 1 | 13 | - | - | - | - | - |
| Chen | 2012 | 49 | 27 | Positive | - | - | - | - | - | - | - | - | - | - | 15 | 12 | - |
|  |  |  |  | Negative | - | - | - | - | - | - | - | - | - | - | 4 | 18 | - |
| Geng | 2008 | 59 | 23 | Positive | - | - | - | - | - | - | - | - | 8 | 15 | 4 | 19 | - |
|  |  |  |  | Negative | - | - | - | - | - | - | - | - | 16 | 20 | 4 | 32 | - |
| Guan | 2014 | 52 | 46 | Positive | 26 | 20 | - | - | - | - | 22 | 24 | - | - | 24 | 22 | - |
|  |  |  |  | Negative | 2 | 4 | - | - | - | - | 2 | 4 | - | - | 0 | 6 | - |
| Guo | 2014 | 98 | 78 | Positive | 46 | 32 | 65 | 13 | - | - | - | - | - | - | 40 | 38 | - |
|  |  |  |  | Negative | 13 | 7 | 9 | 11 | - | - | - | - | - | - | 1 | 19 | - |
| Hu | 2009 | 35 | 23 | Positive | - | - | - | - | - | - | 19 | 4 | - | - | 13 | 10 | - |
|  |  |  |  | Negative | - | - | - | - | - | - | 9 | 3 | - | - | 2 | 10 | - |
| Hu | 2015 | 50 | 29 | Positive | - | - | - | - | - | - | - | - | - | - | - | - | - |
|  |  |  |  | Negative | - | - | - | - | - | - | - | - | - | - | - | - | - |
| Kubo | 2008 | 20 | 8 | Positive | - | - | - | - | - | - | 5 | 3 | - | - | - | - | - |
|  |  |  |  | Negative | - | - | - | - | - | - | 2 | 10 | - | - | - | - | - |
| Li | 2012 | 102 | 47 | Positive | 40 | 7 | - | - | - | - | - | - | - | - | 16 | 31 | - |
|  |  |  |  | Negative | 48 | 7 | - | - | - | - | - | - | - | - | 6 | 49 | - |
| Li | 2015 | 28 | 15 | Positive | 10 | 5 |  | - | 8 | 7 |  | - | - | - | 14 | 1 | - |
|  |  |  |  | Negative | 7 | 6 |  | - | 8 | 5 |  | - | - | - | 7 | 6 | - |
| Lian | 2013 | 35 | 12 | Positive | - | - | - | - | - | - | - | - | - | - | - | - | 28.12 ± 7.84 |
|  |  |  |  | Negative | - | - | - | - | - | - | - | - | - | - | - | - | 12.12 ± 3.86 |
| Luo | 2009 | 51 | 32 | Positive | - | - | - | - | - | - | 26 | 6 | - | - | - | - | 32.2 ± 4.2 |
|  |  |  |  | Negative | - | - | - | - | - | - | 10 | 9 | - | - | - | - | 14.7 ± 2.5 |
| Ma | 2014 | 80 | 40 | Positive | 26 | 14 | - | - | - | - | - | - | 33 | 7 | - | - | 46.85 ± 7.72 |
|  |  |  |  | Negative | 28 | 12 | - | - | - | - | - | - | 29 | 11 | - | - | 28.47±4.31 |
| Mao | 2007 | 64 | 37 | Positive | - | - | - | - | - | - | - | - | - | - | - | - | 32 ± 8 |
|  |  |  |  | Negative | - | - | - | - | - | - | - | - | - | - | - | - | 17 ± 6 |
| Mizobuchi | 2008 | 48 | 18 | Positive | - | - | - | - | - | - | - | - | - | - | 11 | 7 | - |
|  |  |  |  | Negative | - | - | - | - | - | - | - | - | - | - | 8 | 22 | - |
| Naggar | 2012 | 25 | 13 | Positive | - | - | - | - | - | - | 12 | 1 |  | - | - | - | - |
|  |  |  |  | Negative | - | - | - | - | - | - | 3 | 9 |  | - | - | - | - |
| Qian | 2007 | 25 | 14 | Positive | 8 | 6 | - | - | - | - | - | - | - | - | - | - | - |
|  |  |  |  | Negative | 7 | 4 | - | - | - | - | - | - | - | - | - | - | - |
| Wang | 2004 | 46 | 16 | Positive | - | - | - | - | - | - | - | - | - | - | - | - | 38.221 ± 26.935 |
|  |  |  |  | Negative | - | - | - | - | - | - | - | - | - | - | - | - | 29.706 ± 18.694 |
| Wang | 2017 | 103 | 57 | Positive | 23 | 34 | 28 | 29 | 36 | 21 | - | - | - | - | 44 | 13 |  |
|  |  |  |  | Negative | 26 | 20 | 29 | 17 | 19 | 27 | - | - | - | - | 13 | 33 |  |
| Wu | 2010 | 36 | 15 | Positive | - | - | - | - | - | - | - | - | 14 | 1 | - | - | 18.47 ± 8.52 |
|  |  |  |  | Negative | - | - | - | - | - | - | - | - | 13 | 8 | - | - | 10.99 ± 5.54 |
| Yang | 2007 | 39 | 31 | Positive | 11 | 6 | 11 | 6 | 12 | 7 | - | - | - | - | - | - | - |
|  |  |  |  | Negative | 11 | 11 | 17 | 15 | 11 | 9 | - | - | - | - | - | - | - |
| Yin | 2010 | 36 | 22 | Positive | - | - | - | - | - | - | - | - | - | - | - | - | - |
|  |  |  |  | Negative | - | - | - | - | - | - | - | - | - | - | - | - | - |
| Zeng | 2010 | 45 | 25 | Positive | 14 | 11 | 18 | 7 | 14 | 11 | - | - | - | - | 20 | 5 | - |
|  |  |  |  | Negative | 11 | 9 | 12 | 8 | 11 | 9 | - | - | - | - | 5 | 15 | - |
| Zhao | 2015 | 88 | 50 | Positive | 35 | 15 | 41 | 9 | - | - | - | - | 47 | 2 | 17 | 32 | - |
|  |  |  |  | Negative | 34 | 4 | 29 | 9 | - | - | - | - | 33 | 5 | 6 | 33 | - |
| Zheng | 2009 | 30 | 15 | Positive | 10 | 5 | - | - | 8 | 7 | 7 | 9 | - | - | 13 | 2 | - |
|  |  |  |  | Negative | 8 | 7 | - | - | 9 | 6 | 1 | 13 | - | - | 8 | 7 | - |

Annotation: HIF-1α +: hypoxia-inducible factor-1α positive; MVD: microvessel density; -: not reported.
