# Supplementary figures and images for: Clinicopathological and prognostic value of hypoxia-inducible factor-1α in patients with bone tumor: a systematic review and meta-analysis
Source: J Orthop Surg Res. 2019 Feb 19;14:56. doi: 10.1186/s13018-019-1101-5 (PMC6381668; doi:10.1186/s13018-019-1101-5)

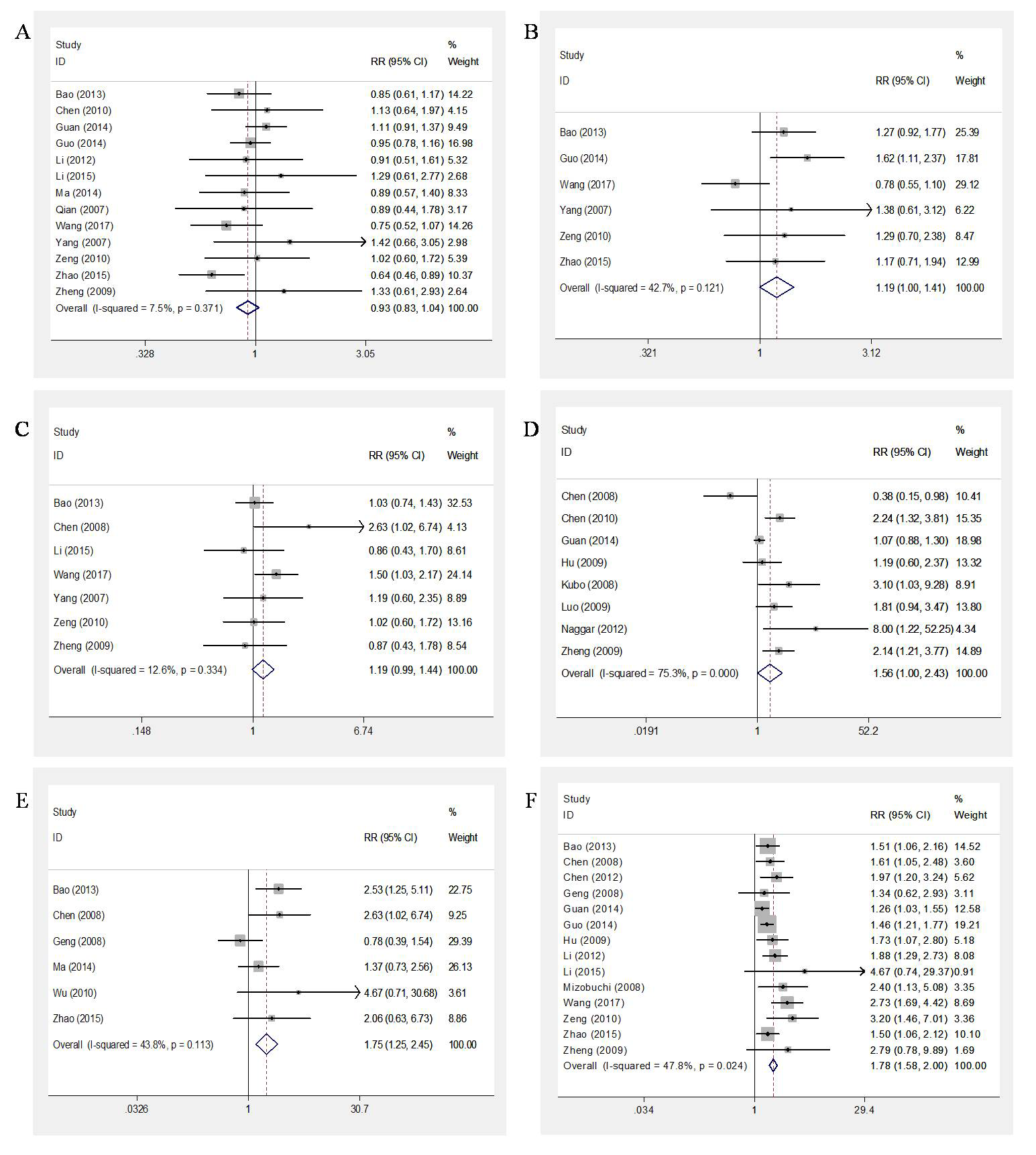

Supplement: Supplementary file 2 — Figure S1. Forest plots of the association between HIF-1α expression and the clinicopathological factors of patients with bone tumor including gender (A), age (B), tumor size (C), differentiation (D), clinical stage (E) and metastasis (F). (TIF 1328 kb) [file 13018_2019_1101_MOESM2_ESM.tif]

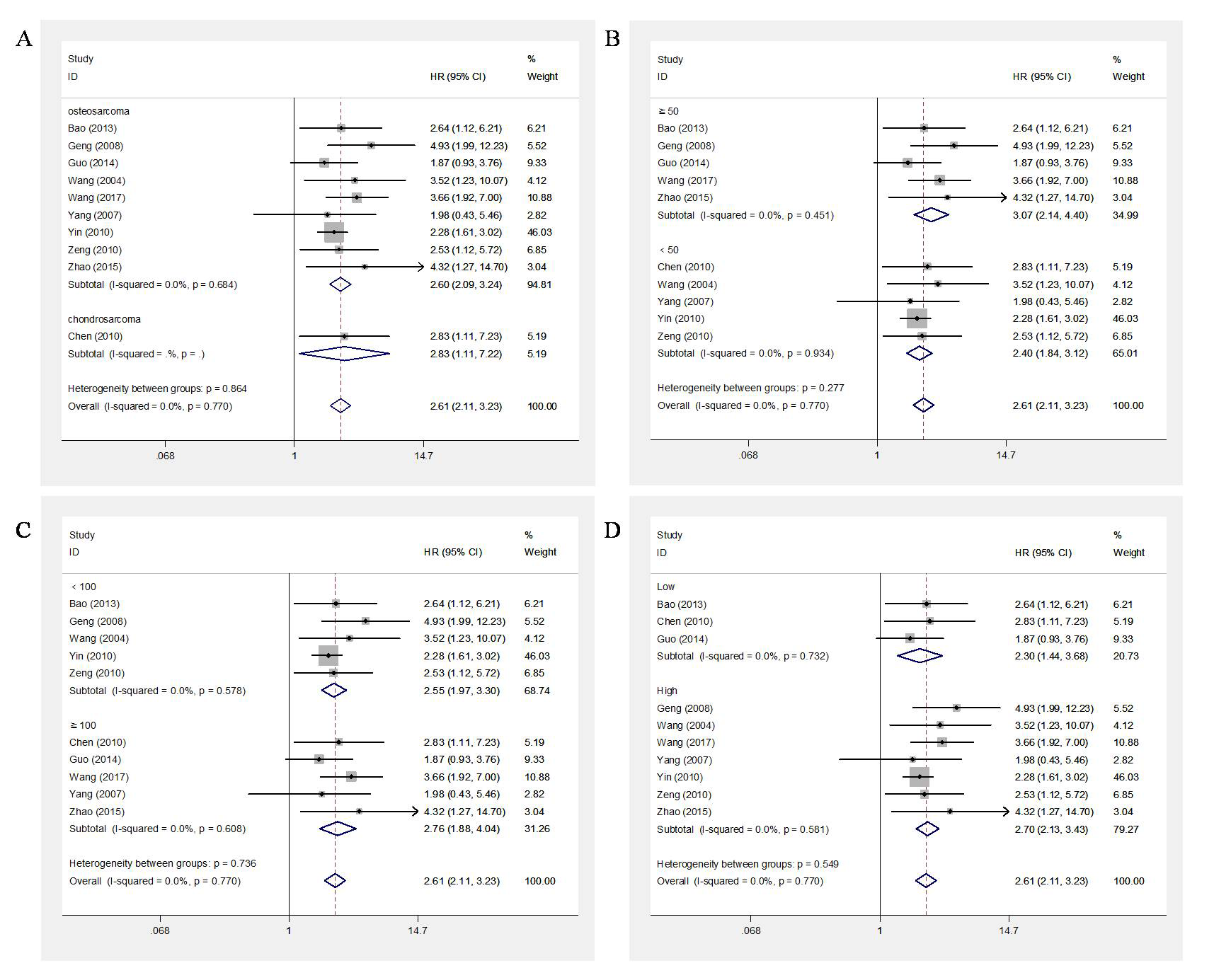

Supplement: Supplementary file 3 — Figure S2. Forest plots of subgroup analyses on the association between HIF-1α expression and OS including histological type (A), the number of case (B), follow-up time (C) and the quality of included articles (D). (TIF 1145 kb) [file 13018_2019_1101_MOESM3_ESM.tif]

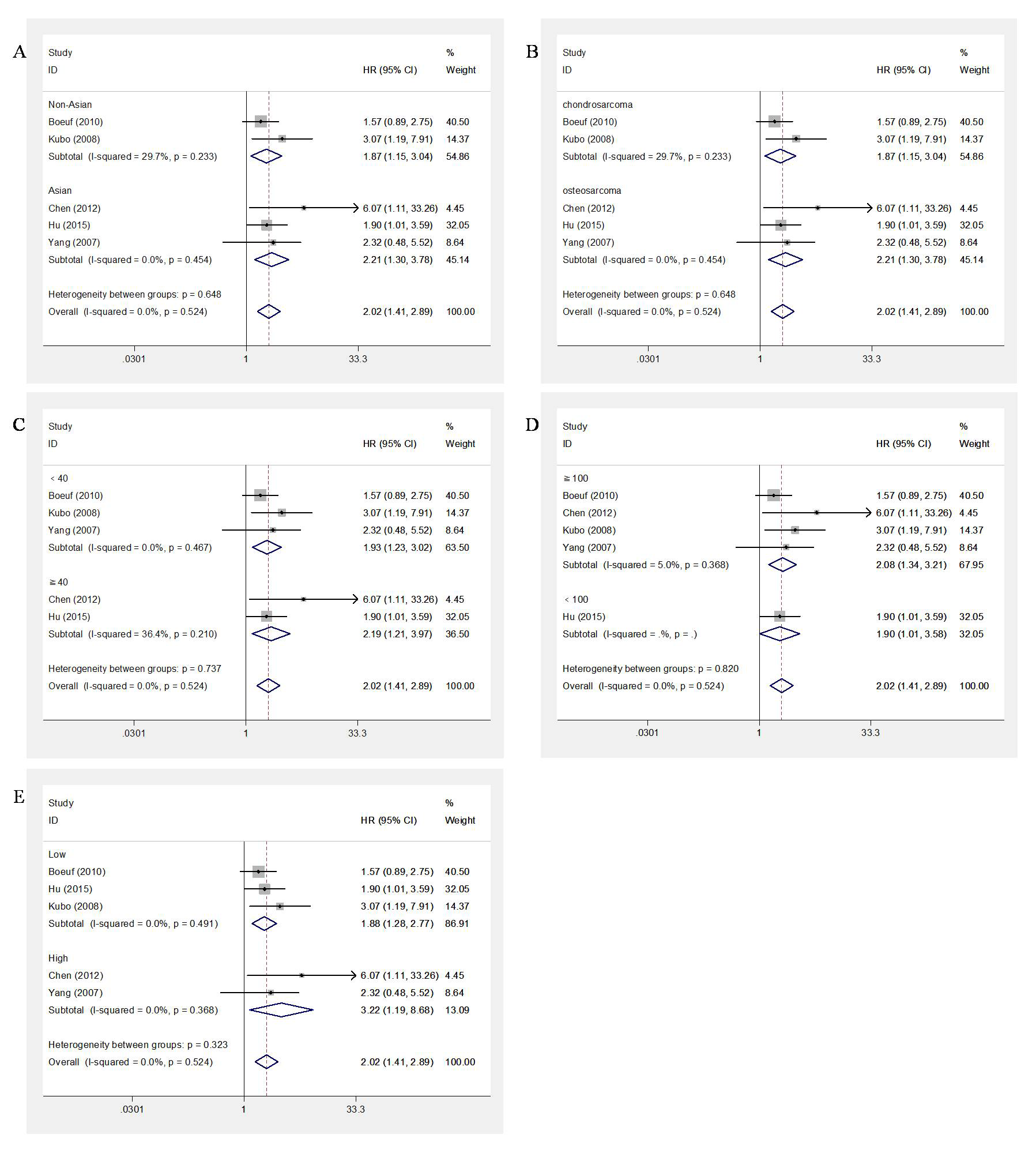

Supplement: Supplementary file 4 — Figure S3. Forest plots of subgroup analyses on the association between HIF-1α expression and DFS including region (A), histological type (B), the number of case (C), follow-up time (D) and the quality of included articles (E). (TIF 1333 kb) [file 13018_2019_1101_MOESM4_ESM.tif]
